# Preoperative tibiofemoral contact point on standing lateral radiograph predicts anteroposterior knee kinematics in total knee arthroplasty

**DOI:** 10.1002/jeo2.70093

**Published:** 2024-11-14

**Authors:** Yusuke Tominaga, Tomofumi Kinoshita, Kazunori Hino, Tatsuhiko Kutsuna, Kunihiko Watamori, Takashi Tsuda, Yusuke Horita, Masaki Takao

**Affiliations:** ^1^ Department of Orthopaedic Surgery Minamimatsuyama Hospital Matsuyama Ehime Japan; ^2^ Department of Orthopaedic Surgery Ehime University Graduate School of Medicine Toon Ehime Japan

**Keywords:** anterior paradoxical movement, knee kinematics, osteoarthritis, tibiofemoral contact point, total knee arthroplasty

## Abstract

**Purpose:**

This study investigated the tibiofemoral contact point (CP) on standing lateral radiographs in knee osteoarthritis and assessed the relationship between CP and pre‐ and postoperative knee kinematics in total knee arthroplasty (TKA).

**Methods:**

The intraoperative knee status of 46 knees with varus deformity that underwent bicruciate stabilized TKA using a navigation system was investigated. The intraoperative anteroposterior (AP) position of the femur relative to the tibia at maximum extension, 15°, 30°, 45°, 60° and 90° was evaluated by the navigation system before and after TKA. The CP, defined as the CP between the femur and tibia, was assessed on standing lateral radiographs at the maximum extension of the knee before and 1 year after TKA. CP was expressed as the ratio of tibial plateau distance on a scale of 0–1, with 0 indicating the most anterior position of the femur relative to the tibia.

**Results:**

The mean CP on standing lateral radiographs was 0.54 ± 0.12 and 0.46 ± 0.08 before and after TKA, respectively. The postoperative CP was significantly more anterior than the preoperative CP (*p* = 0.0002). The mean AP position of the femur relative to the tibia moved anteriorly during early knee flexion both before and after TKA, representing nonanatomical AP movement. The preoperative posterior CP group (CP > 0.54) showed more nonanatomical AP movement from 15° to 60° before and after TKA compared with the preoperative anterior CP group (CP < 0.54).

**Conclusion:**

Preoperative posterior deviation of the femur relative to the tibia in the standing position was a predictive factor for nonanatomical AP knee kinematics. Biomechanical analysis of postoperative knees will be necessary; however, surgeons should focus on preoperative tibiofemoral CP on standing lateral radiographs to predict knee kinematics.

**Level of Evidence:**

Level Ⅲ.

**Abbreviations**
ACL: anterior cruciate ligament
AP: anteroposterior
BCS: bicruciate stabilized
CP: contact point
TKA: total knee arthroplasty

## INTRODUCTION

Total knee arthroplasty (TKA) is an effective treatment for reducing pain and improving quality of life in patients with end‐stage knee osteoarthritis [9]. Despite improvements in modern TKA, solutions are still needed for patients who are dissatisfied with the clinical outcomes after TKA, including functional complications such as limited range of motion and anterior knee pain [2, 5, 10, 18]. Knee kinematics is one of the most controversial factors related to patient satisfaction. Nishio et al. reported that restoration of normal knee rotational kinematics led to good clinical results [17]. However, the postoperative kinematics after TKA present nonanatomical kinematics that differ from those of a normal knee [1, 20, 21] and that nonanatomical rotational kinematics worsen clinical outcomes [14].

Anteroposterior (AP) kinematics have been less extensively validated than rotational kinematics. One study observed nonanatomical femoral movement in several cases, even preoperatively [11]. Dennis et al. reported the incidence of nonanatomical femoral anterior movement following TKA [4]. Such AP kinematics reportedly induce a limited range of motion in the knee joint and worsen clinical outcomes [4, 24]. Despite the importance of AP knee kinematics, the related factors and surgical techniques to control them remain unclear. Hence, a better understanding of AP kinematics may lead to improved postoperative outcomes. Therefore, this study investigated the tibiofemoral contact point (CP) on standing lateral radiographs, which is easy to evaluate before TKA, in patients with knee osteoarthritis, and assessed the relationship between CP and knee kinematics. We hypothesized that the CP on standing lateral radiographs could be a predictor of knee AP kinematics.

## METHODS

This study included patients with osteoarthritis who underwent TKA using a navigation system and was approved by the Institutional Review Board of our university (IRB number: 2309005). Written informed consent was obtained from all patients. This study was performed in accordance with the ethical standards as described in the 1964 Declaration of Helsinki and its later amendments.

**Figure 1:**
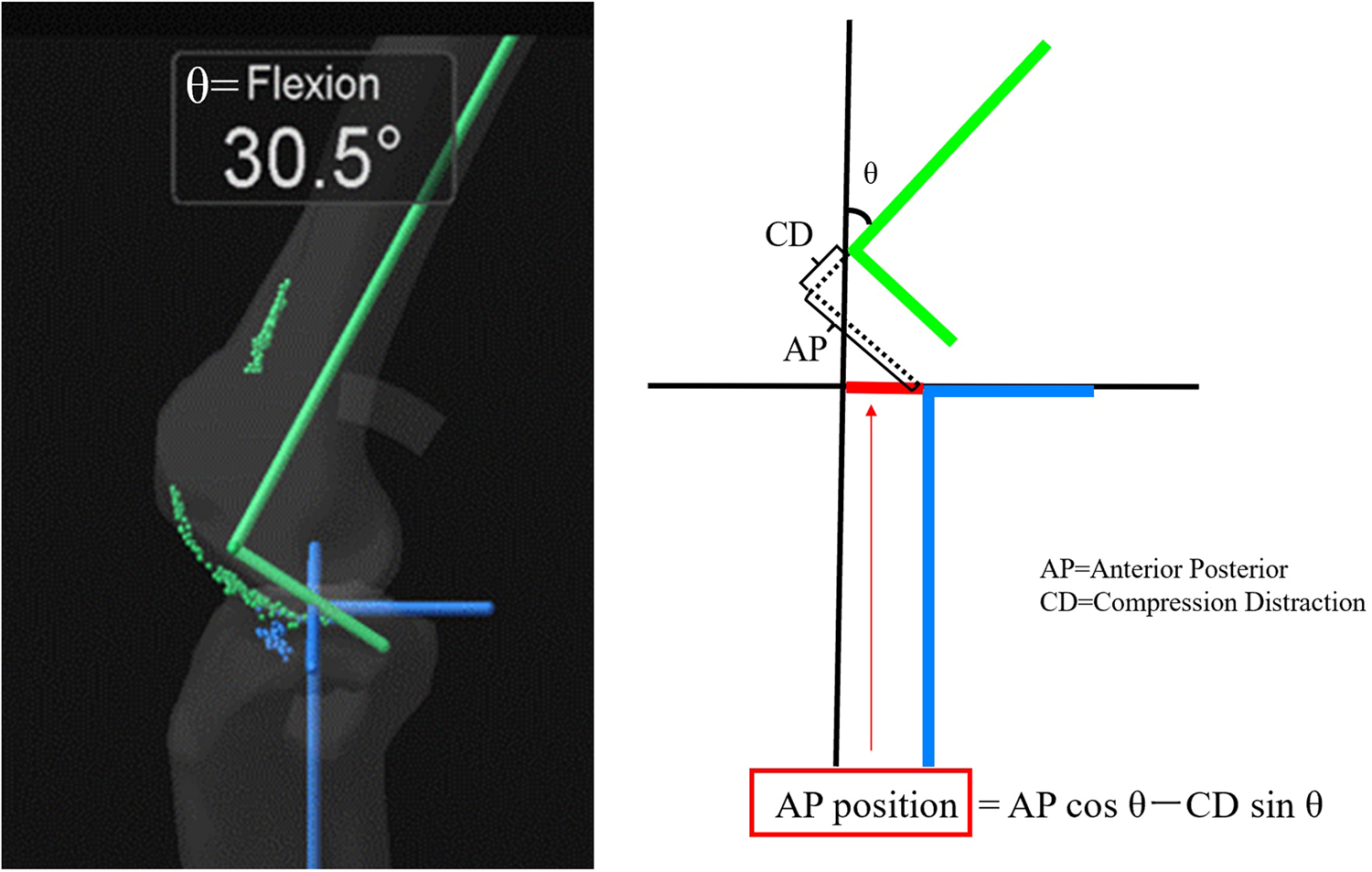
Measurement of the AP position of the femur relative to the tibia. Left image: navigation monitor to evaluate the knee status. Right image: schematic representation of the knee status obtained from the navigation system. The equation is used to calculate the AP distance of the femoral centre relative to the tibial centre based on the parameters obtained from the navigation system. θ, knee flexion angle; AP, anteroposterior distance of the tibial centre relative to the femoral centre; CD, distraction‐compression distance of the tibial centre relative to the femoral centre.

This study analyzed 46 knees from 40 patients (35 females and five males; mean age, 75.7 ± 6.9 [range: 61–87] years) with osteoarthritis who underwent TKA, using a navigation system. To accurately assess and minimize the influence of clinical variables, patients with a history of knee surgery and infection were excluded. Patient characteristics are presented in Table 1. A navigation system (Precision Knee Navigation Software version 4.0, Stryker) was used to evaluate intraoperative knee kinematics. The patients underwent bicruciate stabilized (BCS) TKA (Journey II BCS: Smith & Nephew). The air tourniquet was inflated to 250 mmHg with the patients under general anaesthesia. A 12‐cm skin incision was made to expose subcutaneous tissue. The medial parapatellar approach was used in all cases in this study. Registration was then performed using osteophytes and soft tissues, and the anterior cruciate ligament (ACL) was preserved. Specific anatomical reference points were located by anchoring the infrared signal transducers to the femur and tibia using pins. After registration, the joint capsule was temporarily closed using four suture strands. Mild passive knee flexion was manually applied without angular acceleration while moving the leg from full extension to deep flexion. Subsequently, the AP and compression‐distraction status of the tibia centre relative to the femur centre at maximum extension angle, 15°, 30°, 45°, 60° and 90° were automatically measured using the navigation system. Data were measured every 0.5° or 1 mm. Regarding the AP position of the femur relative to the tibia, we evaluated the femoral centre movement relative to the tibia as previously described [11]. We calculated the AP position of the femur relative to the tibia using the status of the tibia relative to the femur obtained using a navigation system (Figure 1). For the AP status, positive values indicated the anterior, whereas negative values represented the posterior position of the tibia relative to the femur. For the compression‐distraction status, positive values indicated the compression, whereas negative values indicated the distraction position of the tibia relative to the femur. Therefore, the positive and negative signs of the AP and compression‐distraction values changed depending on the position of the femur and tibia. Subsequently, the distal femur was then cut using the measured resection technique. Based on the concept of mechanical alignment, the distal femoral cut was made perpendicular to the mechanical axis of the femur, while the proximal tibial cut was made perpendicular to the mechanical axis of the tibia, and the posterior tibial slope (PTS) was targeted at 3°. After removing the osteophytes, the trial components and trial inserts were placed. After the trial, the components and inserts of proper thickness were placed in the appropriate position with cement and the surgical incision was closed. Subsequently, the kinematics were assessed using the same procedures performed before TKA. In this study, regarding intraoperative evaluation, ‘before TKA’ indicates an assessment immediately after registration using a navigation system, while ‘after TKA’ means an assessment after implant insertion using a navigation system. All data were collected by a single surgeon.

**Table 1:** Pre‐ and postoperative patient characteristics.

	Mean ± SD	
	Preoperative	Postoperative
Hip–knee–ankle angle (°)	12.2 ± 5.1	0.98 ± 2.4
Posterior tibial slope	7.1 ± 2.2	2.6 ± 1.8
Maximum extension (°)	7.2 ± 5.9	0.8 ± 2.1
Maximum flexion (°)	134.9 ± 7.9	128.9 ± 10.9

Abbreviation: SD, standard deviation.

**Figure 2:**
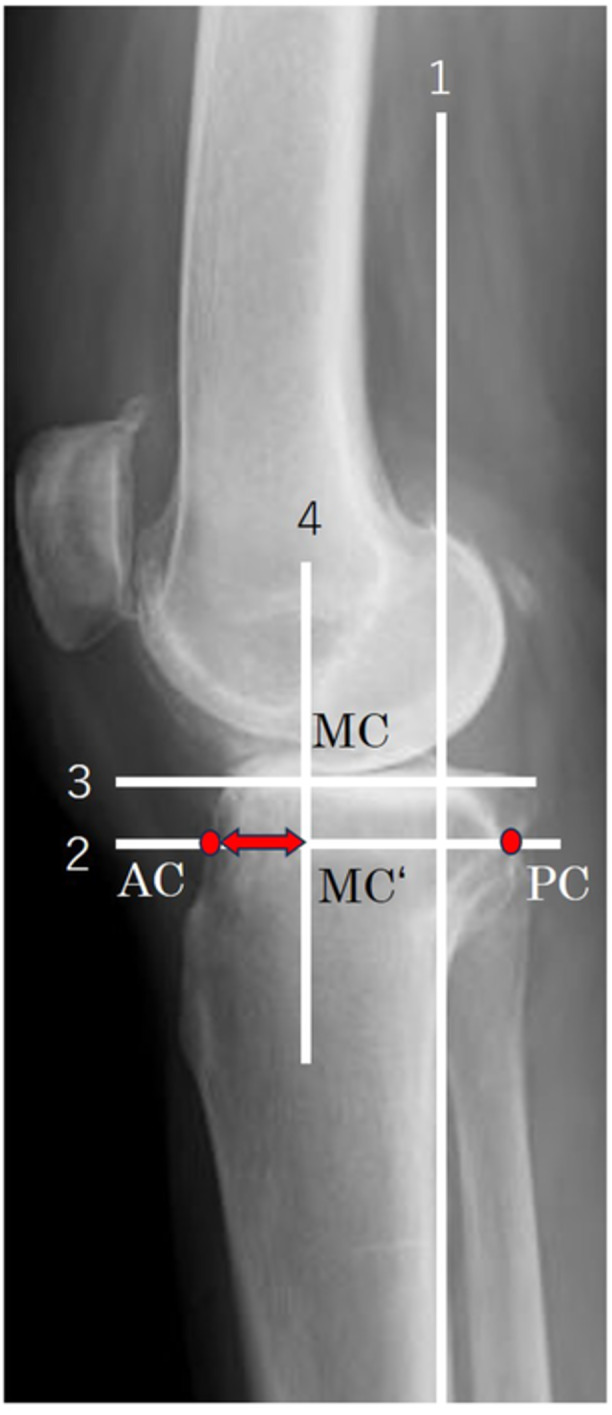
Contact point measurement on standing lateral radiographs. Line 1: a line parallel to the posterior tibial cortex. Line 2: a line 7 mm below the medial tibial plateau and perpendicular to Line 1. Line 3: a line parallel to Line 2 and tangent to the most distal point of the medial condyle (MC). Line 4: a line perpendicular to Line 3 passing through points MC and Line 2. MC′: the crossing between Lines 2 and 4. AC–MC′: the distance between the anterior cortex (AC) and the posterior cortex (PC) of the tibia on Line 2. AC‐PC: distance between AC and PC. The tibiofemoral contact point is calculated as (AC–MC′/AC–PC).

Pre‐ and postoperative tibiofemoral CP on standing lateral radiographs were measured using the method reported by De Jong et al. [3]. We took lateral radiographs at the maximum extension of the knee in a standing position. We ensured that the medial and lateral condyles were overlapping. The CP measurement method is illustrated in Figure 2. A line parallel to the posterior tibial cortex was drawn (Line 1). The medial tibial plateau was identified, and a line perpendicular to Line 1 (Line 2) was drawn 7 mm below the medial tibial plateau. For knees that had undergone TKA, Line 2 was drawn perpendicular to Line 1 at the level of the most distal and posterior edge of the tibial tray. A tangent was drawn parallel to Line 2 to the most distal point of the medial condyle (MC) (Line 3). A line perpendicular to Line 3 was drawn through point MC and Line 2 (Line 4). Point MC′ denotes the crossing between Lines 2 and 4. The anterior cortex (AC) of the tibia on Line 2 was identified, as was the posterior cortex (PC) of the tibia on Line 2. The distance between points AC and PC (distance AC–PC) was measured, as was the distance between points AC and point MC′ (distance AC–MC′). The CP was expressed as the ratio of the tibial plateau distance and calculated as (AC–MC′/AC–PC). The CP was expressed on a scale of 0–1, with 0 indicating the most anterior of the tibia. This method of measurement indicated the medial tibiofemoral CP. To compare the postoperative CP, we have introduced an artificial proximal tibial cut of 7 mm on preoperative radiographs [3]. This adjustment facilitated the comparison of pre‐ and postoperative radiographs as the remaining bony landmarks did not change. The CP on standing lateral radiographs was assessed before TKA and 1 year after TKA.

### Statistical analyses

A power analysis was conducted based on the mean and standard deviation calculated from five preliminary consecutive measurements. The required minimum sample size of 17 was determined to achieve a correlation of δ = 1 and σ = 1, with 80% power and *α* = 0.05, according to the results of the mean difference in the entire landed area's maximum load per body weight between Chair freeze and handstand. Accordingly, we assessed 46 participants to compensate for the small sample size of this study. The Wilcoxon rank‐sum test was used to identify the differences in the knee status of each group. Spearman's rank correlation coefficients (ρ) were used to assess the correlation among the knee status obtained by the navigation system. JMP version 14.0 (SAS Institute) was used for statistical analyses. Statistical significance was set at *p* < 0.05.

## RESULTS

**Figure 3:**
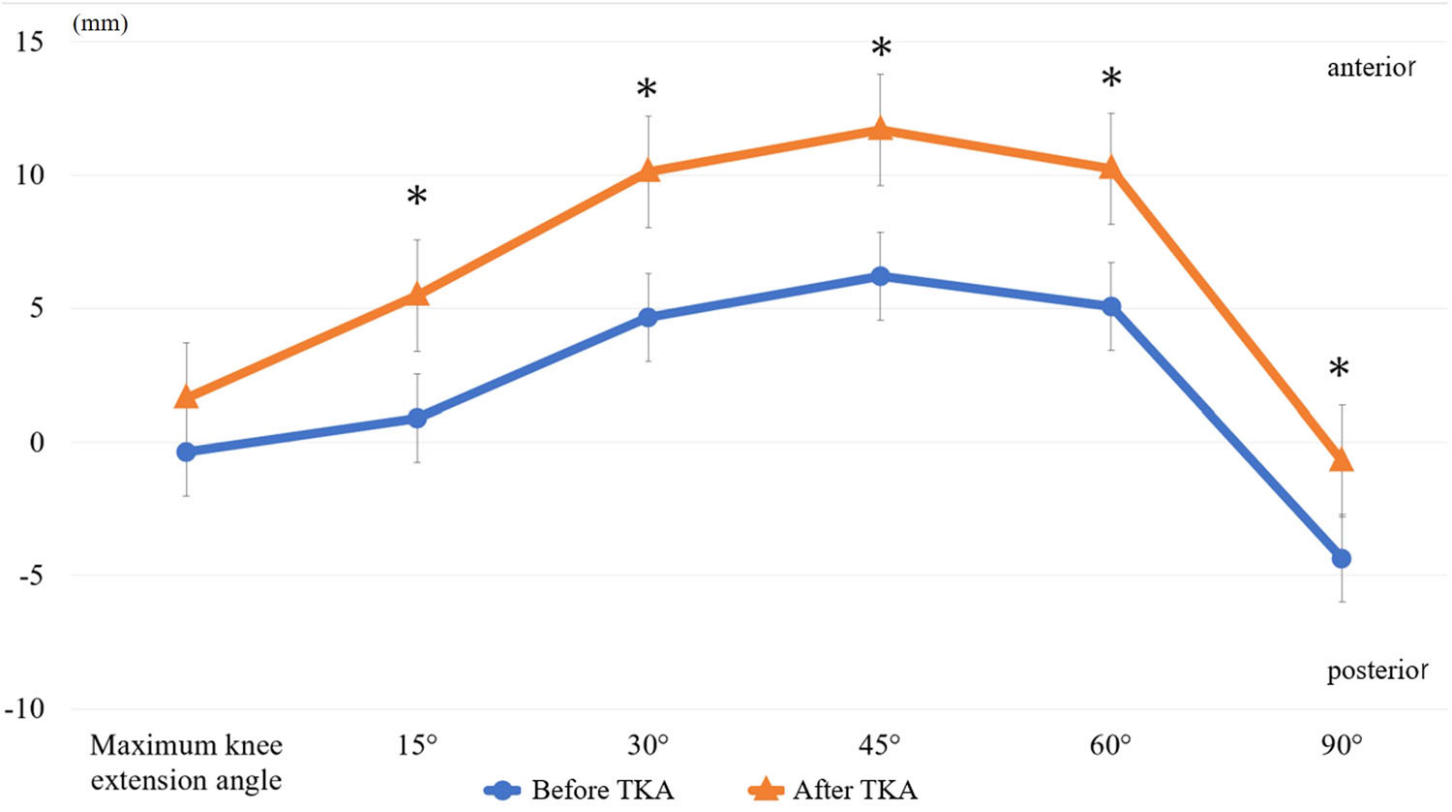
Mean AP translations before and after TKA. Mean AP positions of the femur relative to the tibia at each range of motion before and after TKA. The upper and lower portions of the graph represent anterior and posterior. AP, anteroposterior; TKA, total knee arthroplasty.

Regarding the intraoperative AP position, the mean AP translations before and after TKA are shown in Figure 3. Paradoxical anterior movement was observed during early flexion both before and after TKA. The intraoperative AP position after TKA was significantly anterior at all measured angles compared with the intraoperative AP position before TKA. The intraoperative AP position before TKA was correlated with the intraoperative AP position after TKA, especially during early flexion (Table 2).

**Table 2:** Correlation coefficients between the intraoperative AP positions before and after TKA at each angle.

	*ρ*	*p* Value
Maximum knee extension angle	−0.0243	n.s.
15°	0.3690	0.01*
30°	0.4545	0.002*
45°	0.4651	0.001*
60°	0.3695	0.01*
90°	0.2953	0.04*

Abbreviations: AP, anteroposterior; TKA, total knee arthroplasty.

* *p* < 0.05.

**Figure 4:**
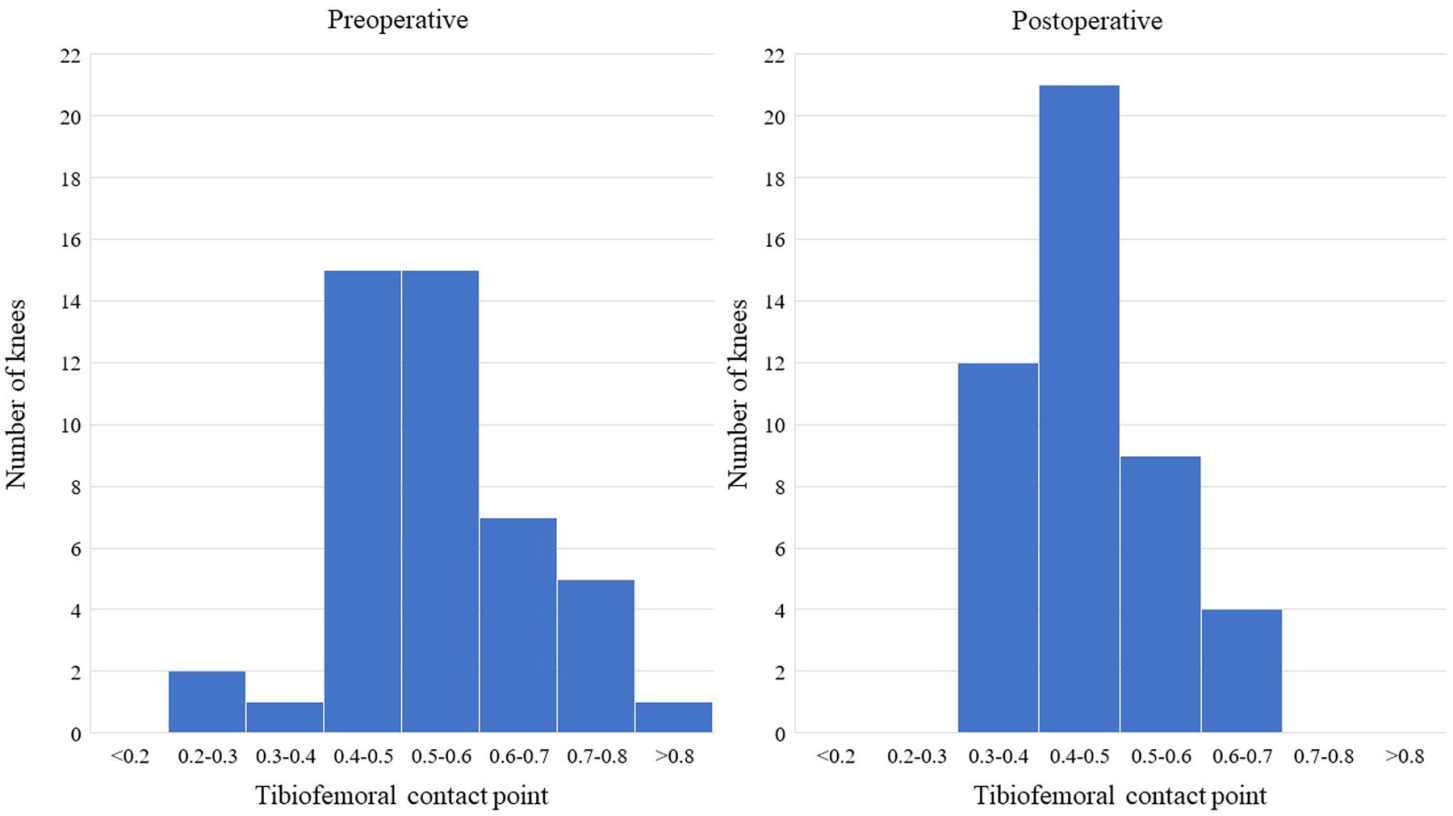
Distribution of pre‐ and postoperative CP on standing lateral radiographs. Left and right graphs: distribution of pre‐ and postoperative CP, respectively. CP, contact point.

The mean CP on standing lateral radiographs was 0.54 ± 0.12 before TKA and 0.46 ± 0.08 after TKA (Figure 4). The postoperative CP was significantly more anterior than the preoperative CP (*p *< 0.001). Pre‐ and postoperative CP showed no significant correlation. The intraoperative AP positions before TKA at maximum extensions, 15°, 30° and 45° were significantly correlated with preoperative CP on standing lateral radiographs (*p* = 0.001, <0.001, 0.002 and 0.03, respectively; Table 3). In contrast, the intraoperative AP position after TKA did not correlate with the postoperative CP (Table 4). Tables 5 and 6 show the correlations among PTS, intraoperative AP positions and CP before and after TKA. Preoperative CP correlated with PTS before TKA (*p* = 0.001); postoperative CP did not correlate with PTS after TKA.

**Table 3:** Correlation coefficients between preoperative CP on standing lateral radiographs and intraoperative AP position before TKA at each angle.

Intraoperative AP position before TKA at each angle	*ρ*	*p* Value
Preoperative CP		
Maximum knee extension angle	−0.5629	0.001*
15°	−0.5153	<0.001*
30°	−0.4470	0.002*
45°	−0.3651	0.03*
60°	−0.2603	n.s
90°	−0.2508	n.s

Abbreviations: AP, anteroposterior; CP, contact point; TKA, total knee arthroplasty.

* *p* < 0.05.

**Table 4:** Correlation coefficients between postoperative CP on standing lateral radiographs and intraoperative AP position after TKA at each angle.

Intraoperative AP position after TKA at each angle	*ρ*	*p* Value
Postoperative CP		
Maximum knee extension angle	0.0113	n.s.
15°	0.0081	n.s.
30°	−0.0326	n.s.
45°	−0.1435	n.s.
60°	−0.0913	n.s.
90°	−0.0480	n.s.

Abbreviations: AP, anteroposterior; CP, contact point; TKA, total knee arthroplasty.

**Table 5:** Correlation coefficients between the preoperative posterior tibial slope and preoperative CP on standing lateral radiographs and intraoperative AP position before TKA at each angle.

	Preoperative posterior tibial slope	
	*ρ*	*p* Value
Preoperative CP	0.5952	0.001*
Intraoperative AP position before TKA at each angle		
Maximum knee extension angle	−0.4318	0.003*
15°	−0.4159	0.004*
30°	−0.3127	0.03*
45°	−0.1052	n.s.
60°	0.0160	n.s.
90°	0.1259	n.s.

Abbreviations: AP, anteroposterior; CP, contact point; TKA, total knee arthroplasty.

* *p* < 0.05.

**Table 6:** Correlation coefficients between the postoperative posterior tibial slope and postoperative CP on standing lateral radiographs and intraoperative AP position after TKA at each angle.

	Postoperative posterior tibial slope	
	*ρ*	*p* Value
Postoperative CP	0.1668	n.s.
Intraoperative AP position after TKA at each angle		
Maximum knee extension angle	0.2758	0.04*
15°	0.2979	0.05*
30°	0.2962	n.s.
45°	0.1649	n.s.
60°	0.1631	n.s.
90°	0.1885	n.s.

Abbreviations: AP, anteroposterior; CP, contact point; TKA, total knee arthroplasty.

* *p* < 0.05.

**Figure 5:**
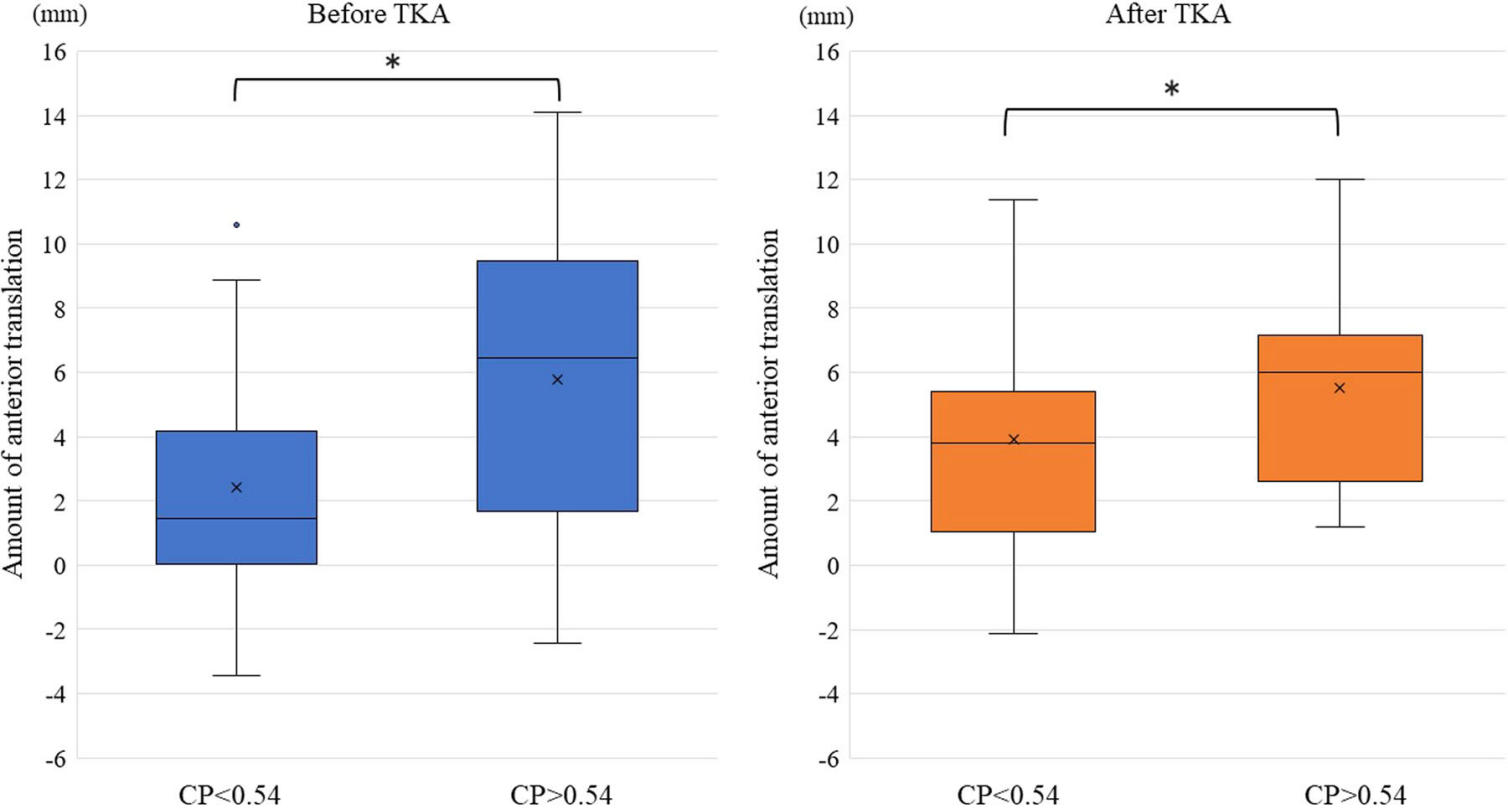
Anterior paradoxical translation from 15° to 60°. Left and right graphs: amounts of anterior paradoxical translation from 15° to 60° before and after TKA, respectively. TKA, total knee arthroplasty.

Finally, all cases were retrospectively divided into two groups according to the preoperative mean CP: preoperative posterior CP group (CP > 0.54) and preoperative anterior CP group (CP < 0.54), with 22 and 24 cases, respectively. The preoperative posterior CP group showed a significantly greater amount of anterior paradoxical translation from 15° to 60° compared with the preoperative anterior CP group, both before and after TKA (*p* = 0.01 and 0.03, respectively; Figure 5).

## DISCUSSION

The important findings of the present study are (1) the correlation between the preoperative CP on standing lateral radiographs and the intraoperative AP positions of the femur relative to the tibia, (2) the influence of preoperative CP on pre‐ and postoperative knee kinematics and (3) the occurrence of anterior paradoxical translation of the femur relative to the tibia during early flexion before and after TKA. In this study, the preoperative CP in the standing position influenced the intraoperative AP translation before and after TKA. The results of this study suggest that preoperative AP malposition in the standing position affects knee kinematics, even after TKA. To the best of our knowledge, no other reports have previously described the preoperative factors related to intraoperative nonanatomical knee kinematics.

The kinematic patterns of normal knees present posterior femoral rollback throughout the entire knee flexion range [16]. In contrast, nonanatomical anterior translation of the femur relative to the tibia during early flexion after TKA has been reported and induces a limited range of motion and worsened clinical outcomes [4]. Such nonanatomical AP kinematics are induced by intraoperative factors, such as implant design and PTS [6, 7]. However, the preoperative‐related factors remain unclear. Thus, surgeons cannot predict the occurrence of nonanatomical AP kinematics before TKA. In the present study, the preoperative posterior CP group showed a significantly greater degree of paradoxical anterior translation during early flexion. Based on these results, preoperative CP in the standing position is related to anterior paradoxical translation. Moreover, postoperative knee kinematics can be predicted using simple radiographic examinations.

Li et al. reported that the CP at full extension was 6.3 ± 1.8 mm anterior to the midline of the tibial plateau in the normal knee [13]. In the present study, the mean CP in the standing position in knees with osteoarthritis was located posterior to the midline of the tibial plateau. Deficiency of the ACL induces posterior deviation of the CP [13]. Mullaji et al. reported a correlation between ACL deficiency and posterior plateau cartilage wear [15]. This degenerative change may induce posterior deviation of the CP. The Journey II BCS instrument used in the present study is a guided‐motion TKA and has anterior and posterior cam mechanisms that provide AP stability. In addition, this procedure has a unique insert design with a concave surface on the medial side and a convex surface on the lateral side. The bottom point of the insert was in the middle of the AP width of the insert on the medial side. Therefore, AP malposition at knee extension before TKA has been reported to fix similarly to a normal knee after BCS‐TKA [22, 23]. In this study, the postoperative CP on standing lateral radiographs was significantly more anterior than the preoperative CP. BCS‐TKA involves fixation of the CP during knee extension under full weight bearing. However, we observed intraoperative nonanatomical knee kinematics after TKA. Owing to the conformity in this guided‐motion TKA, a proper AP position may ensure a standing position in BCS‐TKA. However, under nonweight‐bearing during mid‐flexion, which may make it difficult to demonstrate the effectiveness of the implant design, the AP position and kinematics might be affected by other related factors such as preoperative AP malposition.

Previous studies have demonstrated the effects of preoperative knee kinematics on postoperative knee kinematics. Hino et al. evaluated the change in varus‐valgus kinematics before and after TKA using a navigation system and reported that preoperative knee kinematics influenced postoperative kinematic patterns [8]. Kitagawa et al. also evaluated pre‐ and postoperative kinematics using a single‐plane model image registration technique; the preoperative and postoperative kinematic patterns were similar [12]. Seito et al. reported that preoperative knee deformity and kinematics affect postoperative knee kinematics in TKA [19]. The present study also evaluated intraoperative knee kinematics before and after TKA. Despite the use of guided‐motion TKA, the intraoperative AP positions before TKA at 15°, 30°, 45°, 60° and 90° correlated with those after TKA, and an anterior paradoxical translation was observed before and after TKA. The strength of this study is that it investigated not only the intraoperative AP position but also the CP in the standing position before and after TKA. The results of this study suggest that restoring normal knee kinematics may require the development of surgical techniques and select implants, such as high‐constraint designs, for cases in which the preoperative CP is located posteriorly. However, further studies are needed to clarify these procedures.

This study has some limitations. First, AP kinematics were not evaluated in a weight‐bearing state. Second, because surgery was performed under general anaesthesia, the impact on muscle kinematics could not be assessed. Third, the ACL and the meniscus condition were not investigated. We did not perform a preoperative MRI, and, therefore, have no preoperative assessment. Furthermore, we did not perform a quantitative assessment or evaluation of the strength of the ligaments intraoperatively. The ACL condition may have influenced the AP position of the femur relative to that of the tibia. AP instability was also not evaluated. Although no patients showed excessive AP laxity, the relationship between laxity and kinematics should be verified. Fourth, the quality of the radiograph is important since joint rotation and joint obliquity influence the CP measurement. We ensured that medial and lateral femoral condyles overlapped when lateral radiographs were obtained. Moreover, CP was assessed on standing lateral radiographs at the maximum extension of the knee before and 1 year after TKA; however, the degree of flexion at the maximum extension of the knee may differ for each patient. Finally, the clinical effects of the AP position were not evaluated. The relationships among postoperative factors must be verified before these results can be applied to improve clinical outcomes.

## CONCLUSIONS

Anterior translation of the femur relative to the tibia during early flexion was observed before and after TKA. The preoperative posterior deviation of the femur relative to the tibia in the standing position was related to nonanatomical AP knee kinematics. These findings suggest that surgeons should pay attention to preoperative tibiofemoral CP on standing lateral radiographs to predict intraoperative knee kinematics.

## AUTHOR CONTRIBUTIONS

**Yusuke Tominaga**: Designed the study; collected the data; wrote and edited the manuscript. **Tomofumi Kinoshita**: Conceptualized and designed the study; collected the data; performed statistical analysis; edited the manuscript. **Kazunori Hino**: Collected and analyzed the data. **Tatsuhiko Kutsuna**: Collected and analyzed the data. **Kunihiko Watamori**: Collected and analyzed the data. **Takashi Tsuda**: Collected and analyzed the data. **Yusuke Horita**: Collected and analyzed the data. **Masaki Takao**: Designed the study. All authors read and approved the final manuscript.

## CONFLICT OF INTEREST STATEMENT

The authors declare no conflict of interest.

## ETHICS STATEMENT

This study was approved by the Institutional Review Board of our university (2309005), and written informed consent was obtained from all patients.

## Data Availability

Raw data were generated at Ehime University. Derived data supporting the findings of this study are available from the corresponding author [T. K.] on request.
